# Bibliometric analysis of research topics on blood–brain barrier breakdown and cognitive function over the last two decades (2000–2021)

**DOI:** 10.3389/fnagi.2023.1108561

**Published:** 2023-05-30

**Authors:** Ruirui Zhang, Yiqi Zhang, Tong Wu, Weitian Tian, Jiamei Luo, Yumiao Shi, Diansan Su, Huigang Shu, Jie Tian

**Affiliations:** Department of Anesthesiology, Renji Hospital, Shanghai Jiao Tong University School of Medicine, Shanghai, China

**Keywords:** bibliometric analysis, blood–brain barrier, cognitive function, CiteSpace, VOSviewer

## Abstract

**Introduction:**

Blood–brain barrier (BBB) breakdown is closely associated with cognitive dysfunction. This study aimed to categorize and summarize research topics on the correlation between BBB breakdown and its effects on cognitive function.

**Methods:**

Bibliometric analysis methods were used to quantitatively and qualitatively assess research progress and predict future research hotspots. Relevant publications from the Web of the Science Core Collection were extracted on November 5, 2022 and analyzed to predict trends and hotspots in the field.

**Results:**

We identified 5518 articles published from 2000 to 2021 about the BBB and cognition. The number of manuscripts on this topic increased steadily during this time period, especially after 2013. We found that the number of articles published in China increased gradually and is in second place behind the United States of America (USA). In the research field of BBB breakdown and cognitive function, the USA is still far ahead. Keyword burst detection suggested that cognitive impairment, neurodegeneration disease and neuroinflammation are emerging research hotspots.

**Discussion:**

The mechanisms of BBB integrity breakdown and its effects on the deterioration of cognitive function are complex, and clinical treatment of the affected diseases has been a hot topic in the field over the past 22 years. Looking forward, this body of research is aimed at improving or maintaining patients’ cognitive abilities, by finding preventive measures and to provide a basis for finding new treatments of cognitive disorders.

## Introduction

Under normal physiological conditions, the human brain requires 20% of the cardiac output and consumes 20% of the body’s oxygen and glucose (Iadecola, 2013). Since the brain stores little energy, it obtains energy through its blood supply, which must pass through the blood–brain barrier (BBB; Iadecola, 2017). The BBB is an important structure that maintains the homeostasis of the brain’s internal environment and protects the physiological functions of the central nervous system.

As early as the 20th century, it was discovered that the permeability of the BBB increased with aging, and the integrity of the cerebral blood vessels was crucial for the maintenance of cognitive function during aging (Kalaria, 1996). In-depth research led to a general belief that the BBB is a highly specialized endothelial cell (EC) membrane lining the brain capillaries. It is a barrier system formed by capillary ECs in the brain that are closely connected to each other and interact with the surrounding pericytes and astrocytes. It can regulate plasma components, red blood cells, and white blood cells to enter the central nervous system while ensuring that neurotoxic molecules are exported from the brain to the blood (Abbott et al., 2006; Zlokovic, 2008; Abbott et al., 2010). Microvascular ECs of the brain constitute the main structures of the BBB for several reasons, including their unique tight junctions, lack of openings, and a weak endocytosis and transport effect (Kakinuma et al., 1998). These characteristics can effectively prevent macromolecules from passing through the ECs and, by regulating specific transport proteins, allow nutrients to be transported into the brain and potential toxins to be expelled. In addition, ECs have a low expression of leukocyte adhesion molecules, which prevent immune cells and immune molecules from entering the central nervous system under normal physiological conditions, and they play a certain role in immune surveillance (Obermeier et al., 2013).

If these structures, such as this tight junction, are destroyed as a result of ischemic injury, cerebral hemorrhage, neurodegenerative changes, sepsis, etc., the resulting breakdown of the BBB will lead to the migration and infiltration of immune cells, as well as upset the regulation of the flux of molecules and ions that pass through the BBB. Unhealthy, neurotoxic substances enter the brain, damage synapses and neuronal functions, and cause cognitive impairment and other consequences (Hawkins and Davis, 2005; Zlokovic, 2005). The destruction of the BBB’s integrity is related to the severity of cognitive decline.

Bibliometrics is a discipline that uses mathematics, statistics, and other measurement methods to study the structure and dynamics, quantitative relationships and quantitative management of documents, and to further explore the structure, characteristics, and the laws of science and technology. It is applied mainly through citation analysis, data mining, information visualization, and other methods (Loomes and van Zanten, 2013). Appropriate databases (such as Web of Science or Scopus (Bornmann and Leydesdorff, 2014)) can be used to easily identify and evaluate literature data in various disciplines as well as provide hotspot analysis and trend predictions in current research fields. In this article, we used bibliometric methods to categorize, summarize, and visually analyze relevant literature on the breakdown of the BBB and its effect on cognitive function (BBB-cognition) from 2000 to 2021. We discuss the impact of BBB dysfunction on cognitive function and highlight the mechanisms that contribute to the destruction of the BBB. We describe the effects of the BBB components on cognition, such as junctional complexes, ECs, basal layer, pericytes, and glial cells. We also emphasize the role of aging and beta amyloids and discuss the emerging preventive and therapeutic effects of induced pluripotent stem cells (iPSCs) and their derived technologies in recognizing BBB damage, as revealed by recent data, which provide references that can be used by researchers in the future.

## Materials and methods

We conducted a comprehensive search of the Web of Science Core Collection database using the following search strategies: TS = [((blood–brain barrier) OR (brain blood barrier) OR (hemato encephalic barrier) OR (barrier, hemato encephalic) OR (barrier, blood brain) OR (barrier, brain blood)) AND ((cognition) OR (cognitive function) OR (function, cognitive) OR (memory) OR (learning) OR (executive function) OR (attention) OR (dementia) OR (Alzheimer’s disease))] NOT TI = (guideline or recommendation or consensus or“case report” or meta or review) AND Language = English, with a limited time frame set from 2000 to 2021. To reduce the bias caused by frequent updates of the database, we completed all literature searches and data downloads within 1 day on May 1, 2022.

Two researchers (RZ and YZ) conducted the primary data search independently. Manuscripts were screened and recorded for titles, authors, countries, institutions, journals, and the total/average citation numbers. After discussing potential differences, the final agreement reached 0.90, which was substantially in accordance. Data from the Web of the Science Core Collection were converted to txt format and imported into the Online Analysis Platform of Bibliometry,^1^ CiteSpace V5.6.R5 SE, 64bit (Drexel University, Philadelphia, PA, United States) for further bibliometric analyses. The selection used a modified gindex in each slice: g2 ≤ k Σi ≤ g ci, k ∈ Z+, k = 25.

We intended to describe all literature characteristics, including countries/institutions, journals, highly cited articles, clustered networks of co-cited author, and keywords with the strongest citation bursts. We obtained the impact factors and category quartiles from the Journal Citation Reports 2022, a vital indicator to measure the scientific value of research. Annual publication numbers and growth tendencies were analyzed using the online bibliometric platform. We applied CiteSpace software to visualize the bibliometric data to evaluate collaborations and keyword analyses. For institutional analysis, “Institution”was chosen in the Node Types parameter area, and the rest of the settings were left as default values. For co-authorship network analysis, “Author”was chosen in the Node Types after importing data into CiteSpace. For keywords burst detection, “Keywords”was chosen as the Node Type. Again, “Cosine”was used to calculate the burst strength. After removing keywords with little real significance (like cells, mice, etc), the top 20 keywords with the strongest burst strength were identified and are displayed.

## Results

### Bibliometric analyses of publication output

To describe the relationship between BBB breakdown and cognitive function, rather than discussing the BBB or cognition separately, we looked through all the publications identified by our search strategy. After removing unmatched publications, 5,581 articles remained in the final analysis.

**Figure 1:**
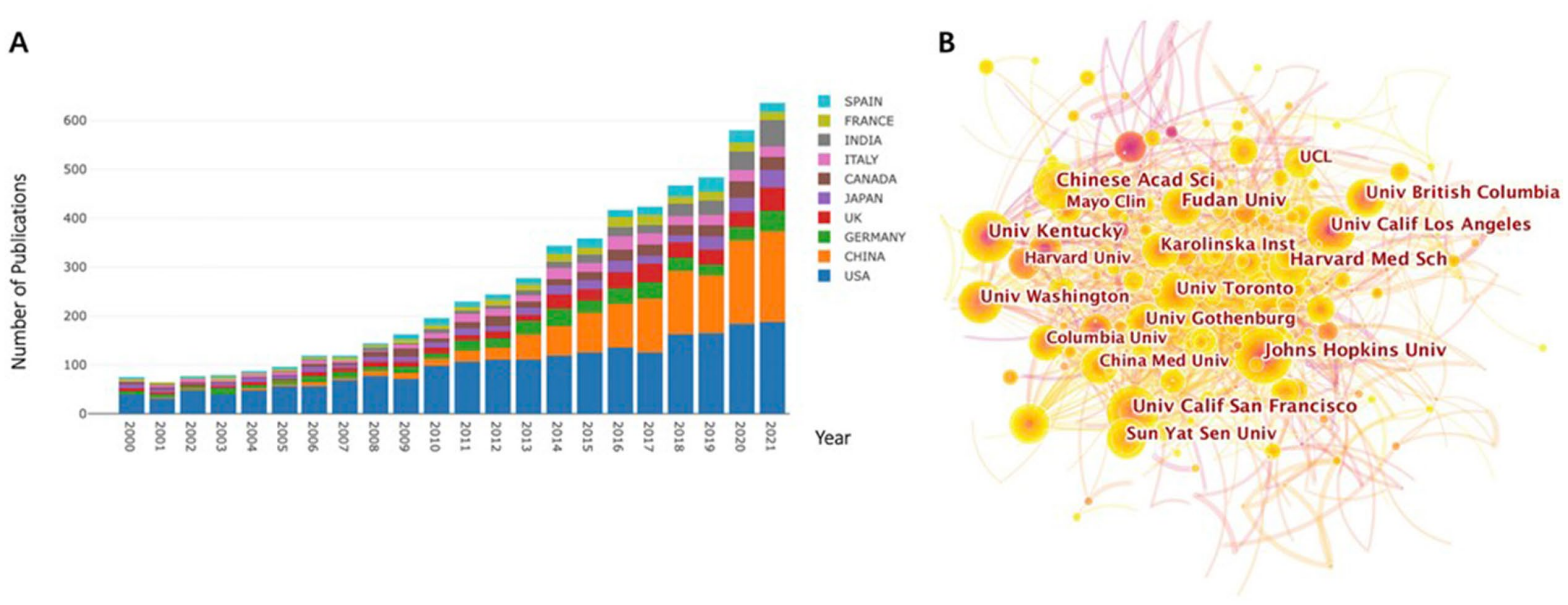
Number of annual publications and growth trends in BBB-cognition research in years 2000 to 2021, displayed by country/regional distribution **(A)** and institutions **(B)**. The results are exported from the Online Analysis Platform of Literature Metrology (http://biblimetric.com). Most publications were from the United States, China was ranked second, followed by Germany, the United Kingdom, and Italy.

We used bibliometric diagrams to visualize the development of a relationship between the BBB and cognition from 2000 to 2021. A trend of increasing numbers of articles in this field was clearly revealed. Moreover, the years 2014, 2016, 2018, and 2020 showed a leap of increases in the number of publications (Figure 1A). The research output shows a broad range of approaches for studying BBB breakdown and cognition.

### Distribution of countries/regions and institutions

Publications originated from 107 countries. The country with the greatest number of publications was the United States, with a total of 2,186 articles over the past 22 years (Figure 1A). China’s contribution to the research field of BBB breakdown and cognitive function was ranked second (1,114 articles), followed by Germany (406 articles), the United Kingdom (352 articles), and Italy (279 articles).

A total of 1,647 institutions were involved in studying this field. The leading research institution with the highest number of publications was Johns Hopkins University (67 articles), followed by the University of Kentucky (62 articles), University of California San Francisco (60 articles), Chinese Academy of Sciences (59 articles), and Fudan University (56 articles; Figure 1B; Supplementary Table S1). Three of the top five institutions were from the United States, suggesting that the United States plays a significant role in this field.

### Journal, co-authorship and high-cited articles network analyses

5,581 manuscripts were published by 1,277 different journals between 2000 and 2021. Table 1 lists the top 10 journals with the highest number of total citations. The highest-ranking journal was Journal of Alzheimer’s Disease, which featured 561 total citations with an average of 2.74 citations per paper, followed by the Journal of Neuroscience and Nature Medicine; the Nature Medicine features the highest impact factor. The Web of Science showed that 7 of the top 10 journals were ranked in Q1, except for the Journal of Alzheimer’s Disease, Neurobiology of Aging and Brain Research, which were all ranked in Q2.

**Table 1:** Top 10 active journals with the highest number of total citations that published articles in BBB-cognition research (sorted by count).

Rank	Journal title	Total citations	Average citation per paper	Impact factor (2021)	Country	JCR
1	Journal of Alzheimers Disease	561	2.74	4.47	Netherlands	Q2
2	Journal of Neuroscience	444	6.25	6.17	the United States	Q1
3	Nature Medicine	422	46.89	53.44	the United States	Q1
4	Neurobiology of Aging	422	46.89	4.67	England	Q2
5	Journal of Clinical Investigation	318	35.33	14.81	the United States	Q1
6	Neuron	311	51.83	17.17	the United States	Q1
7	PLOS ONE	291	2.17	3.24	the United States	Q2
8	Proceedings of the National Academy of Sciences of the United States Of America	290	6.59	11.21	the United States	Q1
9	Journal of Cerebral Blood Flow and Metabolism	279	4.73	6.20	the United States	Q1
10	Brain Research	245	3.18	3.25	Netherlands	Q2

**Figure 2:**
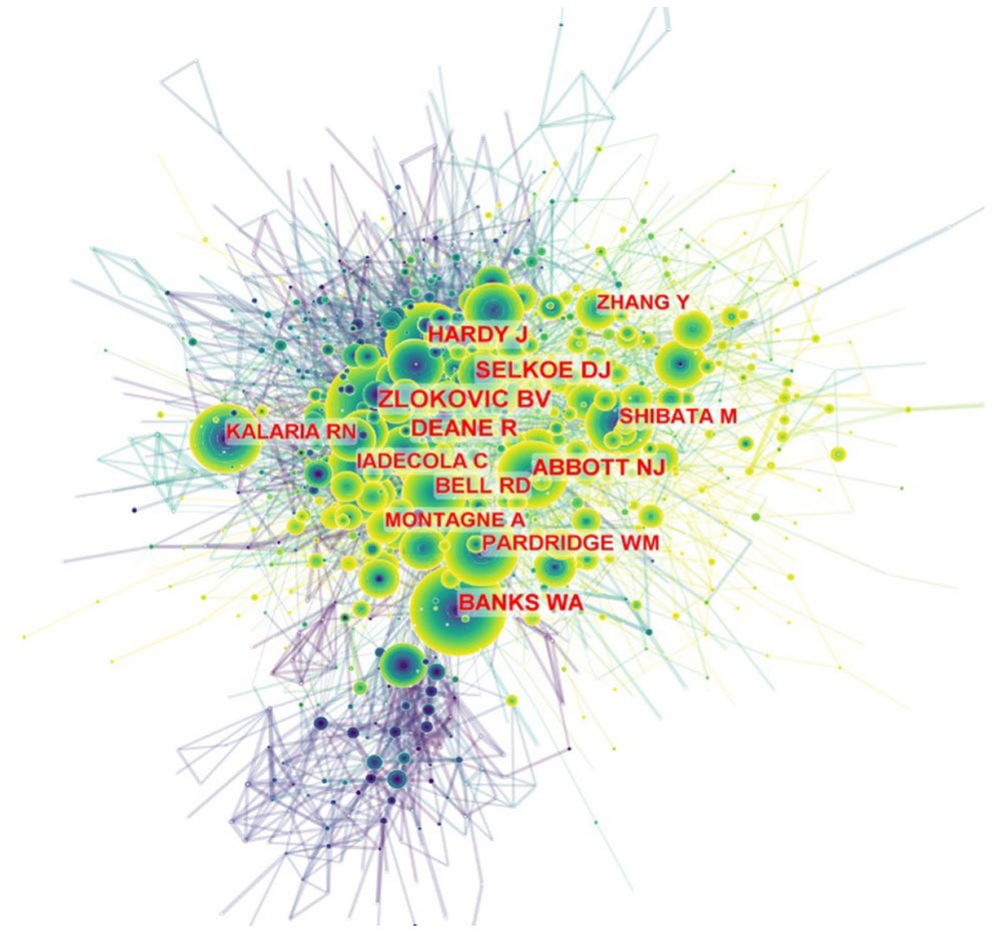
Citespace network of co-cited authorship in the field of BBB and cognition. Every circle represents one author. Size of circle is positively linked to cited counts of the authors, links between two circles represents a collaboration between two authors on the same article. Frequency of collaborations were presented by line thickness. Timespan: 2000 to 2021.

We performed cited-author analyses to list the most cited authors in this field, which is shown in Figure 2; Supplementary Table S2. A co-citation relationship among authors was established when two (or more) authors are meanwhile cited in one or more papers. Using CiteSpace, the co-cited author analyses show 1,255 nodes and 9,731 links. Berislav V. Zlokovic from the Department of Physiology and Neuroscience, Zilkha Neurogenetic Institute, University of Southern California has been cited 727 times in 2001, ranked first among the authors. And ranked second was Dennis J Selkoe from Ann Romney Center for Neurologic Diseases, Department of Neurology, Brigham and Women’s Hospital and Harvard Medical School, Boston, MA, United States. The rest of the major authors are presented in Figure 2.

Table 2 lists the top 10 high-cited articles (Cited Frequency is used as the evaluation criterion, which denotes the frequency of this article cited by the manuscripts in this field. Total Citations means the cited amount of the article in all fields). The research article “Blood–brain barrier breakdown in the aging human hippocampus” published in *Neuron* featured the highest number of citations (140 citations), followed by “The blood–brain barrier in health and chronic neurodegenerative disorders.” published in *Neuron* (93 citations). The third most cited article entitled “Blood–brain barrier breakdown is an early biomarker of human cognitive dysfunction” with 87 citations was published in *Nature Medicine*. These articles are considered fundamental in research on the BBB breakdown and cognitive function.

**Table 2:** Top 10 high-cited articles in BBB-cognition research during the years 2000 to 2021.

Rank	Authors	Years	Journal	Cited frequency	Total Citations	Title
1	Montagne A	2015	Neuron.	140	1,106	Blood–brain barrier breakdown in the aging human hippocampus.
2	Zlokovic BV	2008	Neuron.	93	2,337	The blood–brain barrier in health and chronic neurodegenerative disorders.
3	Daniel A Nation	2021	Nature Medicine.	87	662	Blood–brain barrier breakdown is an early biomarker of human cognitive dysfunction
4	Bell RD	2009	Acta Neuropathologica.	59	676	Neurovascular mechanisms and blood–brain barrier disorder in Alzheimer’s disease.
5	Mawuenyega KG	2010	Science.	55	1,527	Decreased clearance of CNS beta-amyloid in Alzheimer’s disease.
6	Bell RD	2012	Nature	55	869	Apolipoprotein E controls cerebrovascular integrity via cyclophilin A.
7	van de haar HJ	2016	Radiology	53	319	Blood-Brain Barrier Leakage in Patients with Early Alzheimer Disease.
8	Sevigny J	2016	Nature	50	1,618	The antibody aducanumab reduces Aβ plaques in Alzheimer's disease.
9	Matthew R Halliday	2016	J Cereb Blood Flow Metab.	48	341	Accelerated pericyte degeneration and blood–brain barrier breakdown in apolipoprotein E4 carriers with Alzheimer’s disease
10	Deane R	2004	Neuron.	41	703	LRP/amyloid beta-peptide interaction mediates differential brain efflux of Abeta isoforms.

### Keyword

co-occurrence cluster analyses of hotspots and clustered network visualization

**Figure 3:**
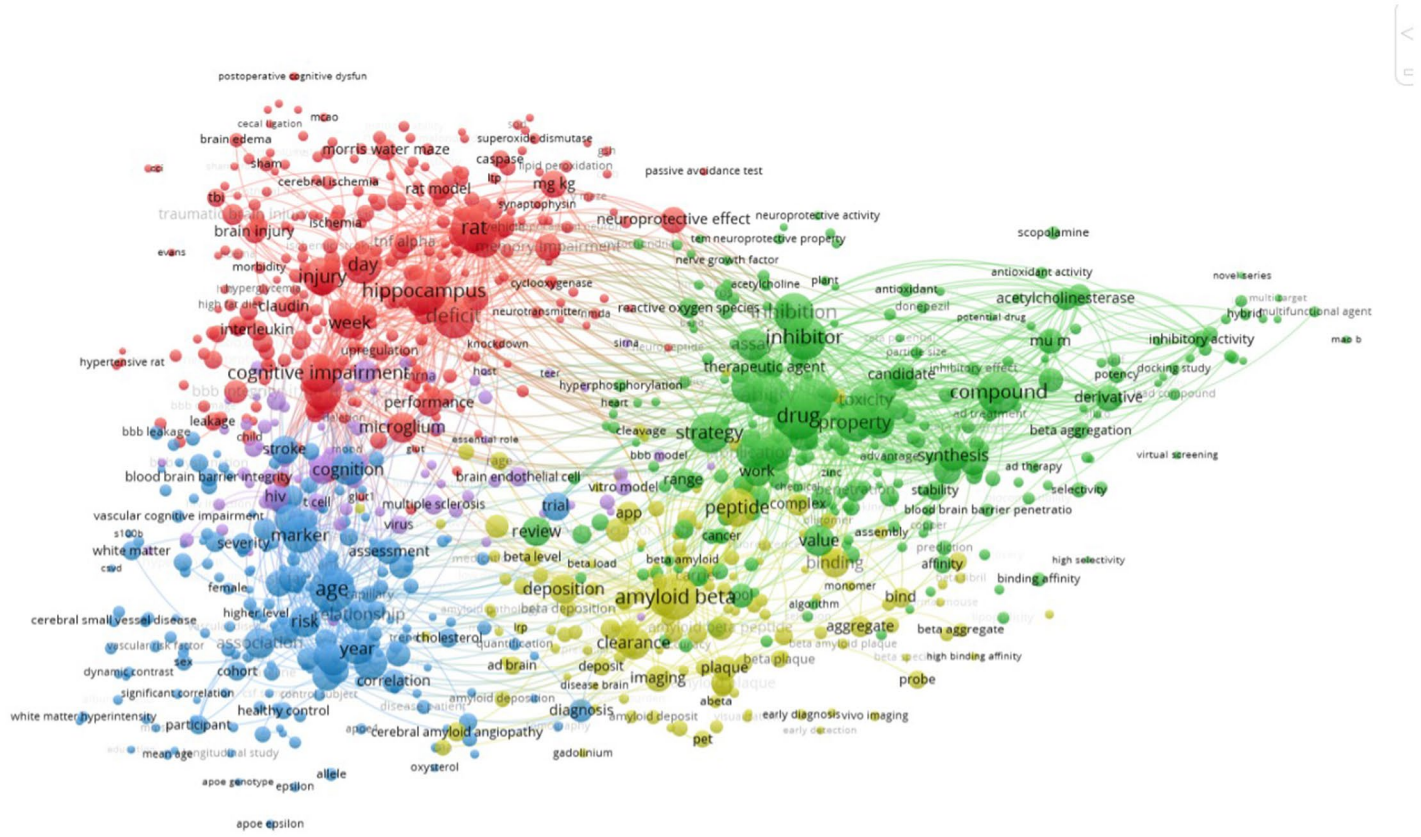
Network map of keyword clustering showing keywords with a minimum occurrence of 20 times, classified into 5 clusters: cognitive impairment, amyloid beta, cognition, age and drug.

Keyword co-occurrence cluster analysis can figure out the research trends on BBB and cognition. To extract keywords from titles and abstracts of the manuscripts included in this study, the Vosviewer software was used. We identified 93 keywords that occurred at a minimum of 20 times and visualized these data using a bubble map. Keywords were classified into five clusters: cognitive impairment, amyloid beta, cognition, age, and drug (Figure 3).

### Burst detection with keywords

**Figure 4:**
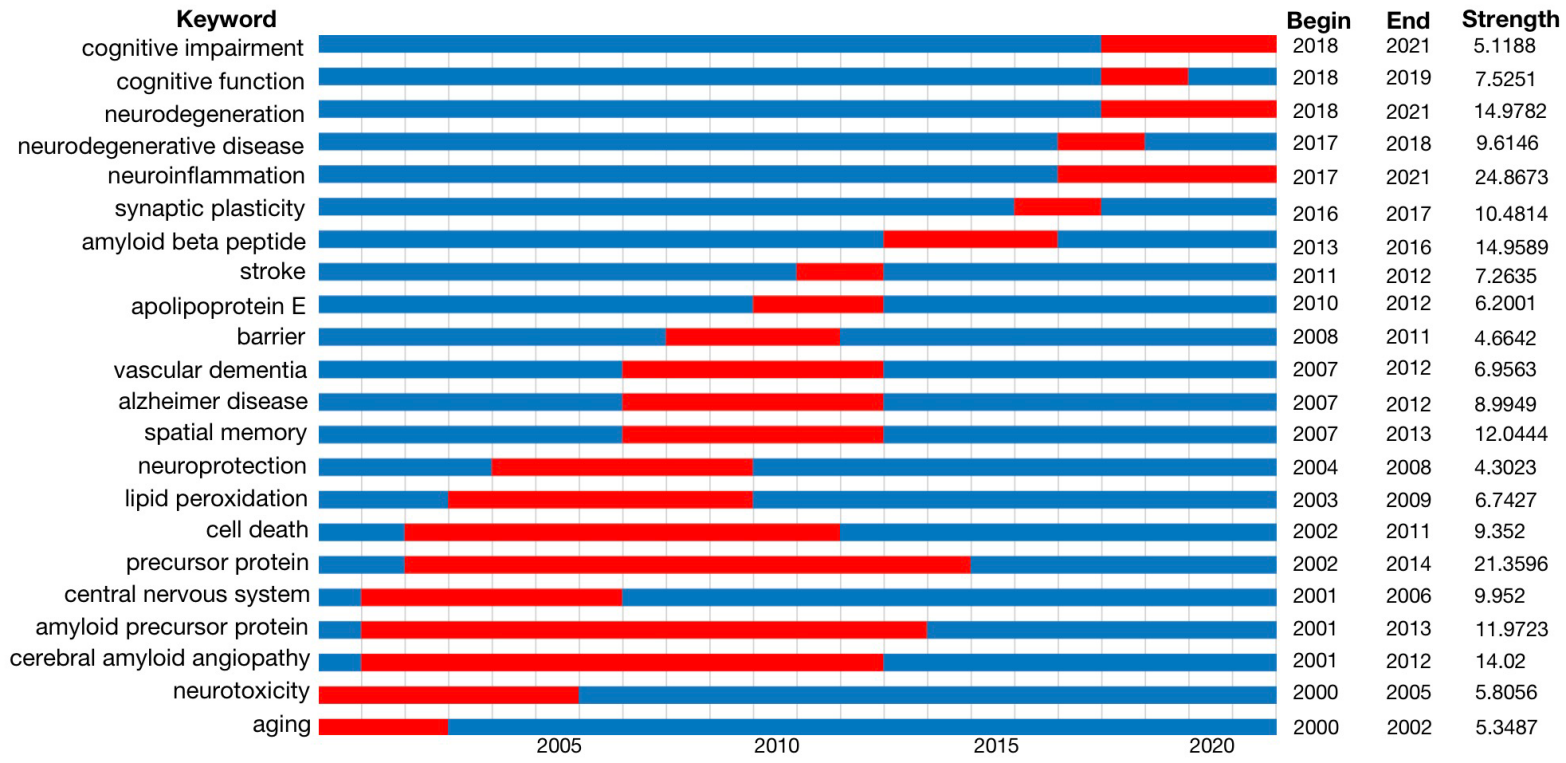
Keywords with the strongest citation bursts in original articles on BBB-cognition research between 2000 and 2021. Keywords marked in red indicates a sudden increase in the usage frequency of this keyword during that period. Blue represents a relatively unpopular time period. It shows that cognitive impairment, neurodegeneration disease and neuroinflammation have been hot topics in recent years in BBB-cognition.

Burst detection is a method to identify concepts that attracted the attention of peer investigators during a certain period of time. We detected a keyword burst in our analysis of the 5,581 articles studied and published over the last 22 years. Figure 4 shows the range of years examined with the duration of citation bursts depicted as red zones. We excluded keywords with little or no research significance and displayed the keywords that represented research trends in BBB breakdown and cognitive function. This keyword burst detection concluded that precursor protein, amyloid precursor protein and cerebral amyloid angiopathy were the most popular topics before 2015, whereas cognitive impairment, neurodegeneration disease and neuroinflammation have been hot topics in recent years.

## Discussion

### Research progress in neurodegenerative diseases and BBB –cognition

Studies of the BBB and cognitive impairment have proven that BBB dysfunction plays an important role in the pathophysiological mechanism of cognitive impairment and dementia (Montagne et al., 2015; Hainsworth et al., 2017; Nation et al., 2019; Montagne et al., 2020). George Glenner first proposed in 1979 that damage to the BBB is one of the pathogenetic mechanisms of neurodegenerative diseases, and since then, AD and other neurodegenerative diseases have attracted widespread attention (Jiang et al., 2018). Study reveals more than 90% of patients with AD have an impaired BBB (Calsolaro and Edison, 2016). Moreover, BBB dysfunction may trigger and/or contribute to a “vicious cycle” of BBB breakdown, due to the disruption of tight connections resulting in altered molecular transport between the brain and blood, vascular degeneration or abnormal angiogenesis, cerebral insufficiency, and inflammatory responses (Zlokovic, 2008).

The development of neurodegenerative diseases is often accompanied by neuroinflammation (Buendia et al., 2016; Calsolaro and Edison, 2016; Ransohoff, 2016; Hickman et al., 2018), which involves the activation of microglia and astrocytes, the invasion of T cells, and the secretion of various inflammatory mediators and nerve cell anti-inflammatory neuropeptides (Laurent et al., 2017; Zhang et al., 2018). Although acute neuroinflammation can have a positive role in repairing CNS damage by enhancing the repair of neurons, if acute inflammation persists and turns into chronic neuroinflammation, it subsequently activates glial cells to produce a large number of inflammatory mediators, which increases oxidative stress on neurons and leads to neuronal cell damage. The BBB is responsible for protecting the central nervous system (CNS) from the peripheral immune system; however, in many neurodegenerative diseases, such as AD, the integrity of the BBB is compromised by cytokines released by inflammation in the CNS, including TNF-α, IL-6, and IL-1β. This damage to the BBB leads to leukocyte migration into the brain, which can exacerbate inflammation in the CNS, creating a vicious circle that contributes to the progression of neurodegenerative diseases. In this process, a variety of leukocytes are involved, including regulatory *T* cells, which are crucial for regulating innate and adaptive immunity. Studies have also shown that Th17 cells can destroy the BBB, exposing a large number of neurons to the peripheral immune system and causing a strong cytotoxic effect, and ultimately leads to the development of cognitive impairment. Therefore, understanding the mechanisms behind neuroinflammation and the BBB is crucial for developing effective therapies for neurodegenerative diseases.

### Research progress in aging and BBB-cognition

The keyword map shows that at the beginning of the 21st century, aging was one of the most prominent keywords to study the connection between the BBB and cognition. One of the main health challenges of aging is a decline in brain cognitive function, including vascular and neurodegenerative dementia, such as AD or PD as mentioned above (Costea et al., 2019). Studies have shown that neurodegenerative diseases related to age-related cognitive dysfunction are mechanically linked to the functional state of the capillaries in the brain (Brown and Thore, 2011). In 2009, Farall and Wardlaw systematically reviewed the literature proved that the permeability of the BBB increased in patients with cerebral microvascular disease. They also determined that the permeability of the BBB increased with age, which may be an important mechanism for the occurrence of cerebral microvascular diseases during aging (Farrall and Wardlaw, 2009). In addition, studies in mice show that the age-dependent progressive loss of pericytes can lead to the destruction of the BBB, resulting in microvascular degeneration, which in turn leads to neurological dysfunction, cognitive decline, and neurodegenerative changes (Bell et al., 2010). Another study demonstrates that expression of the tight junction proteins that compose the BBB, such as claudin-5, was decreased in the brains of both old mice and humans when compared to healthy young control groups; and the permeability of the BBB to 3-kDa glucan was also increased in old mice (Stamatovic et al., 2019).

### Research progress of molecular mechanisms in BBB-cognition

our analyses revealed that the beta amyloid peptide, apolipoprotein E and precursor proteins are hotspots in research, indicating that molecular mechanisms of BBB and cognition deserve more attention. Since Gianvito Martino used iPSCs in mouse models of multiple sclerosis in 2013 (Laterza et al., 2013), iPSC-derived two-dimensional cell models, three-dimensional (3D) organoids, and BBB chips have been used as *in vitro* models to study a variety of neurodegenerative diseases (Logan et al., 2019; Vatine et al., 2019), and these technologies provide novel insights into molecular and genetic mechanisms of diseases of the human brain. For example, in 2021, Yoojin Shin *et.* developed a physiologically relevant model of microfluidic 3D human neural cells, which has a BBB-like phenotype with a brain EC monolayer (Shin et al., 2019), and they found tight junctions in the AD model. They also found an increased expression of protein claudin-5 and the adhesion junction protein VE-cadherin. Future developments of iPSC technology, and other improvements in cell, molecular, and developmental neurobiology, will become driving forces, accelerating the exploration of therapeutic strategies for BBB damage that causes human cognitive impairment disorders.

## Conclusion

Currently, there are exciting developments in the field of BBB and cognition, and our understanding has greatly improved in the last 22 years. Current research focuses on neurodegenerative diseases, which is essential to actively transform our knowledge base from observing research phenomena to exploring mechanisms, eventually resulting in clinical applications, by designing treatments to protect the BBB from deterioration, and by improving the selectivity of the BBB permeability, hereby maintaining cognitive function. We firmly believe that ongoing research on the BBB and cognition will change the face of neuromedicine. Thousands of patients around the world will eventually benefit from previous investigators’ understanding of how the BBB affects cognition, and from which molecular mechanisms are at play in neurological diseases. Bibliometric diagrams greatly assist in describing the overall structure of a body of scientific research on particular topics, such as the correlation between BBB breakdown and cognitive function discussed here, and they provide valuable information for researchers.

## Data availability statement

The original contributions presented in the study are included in the article/Supplementary material, further inquiries can be directed to the corresponding author.

## Author contributions

RZ and YZ contributed equally to the conception of the study, searched, screened, did the data extraction of the studies, drafted, and reviewed the manuscript. JL, YS, and WT contributed to the conception of the study, reviewed, and edited the manuscript. JT and HS conceived the idea of the study, critically reviewed, and edited the manuscript. All authors contributed to the article and approved the submitted version.

## Funding

This work was supported by the National Natural Science Foundation of China (No. 82171177 to JT) and Shanghai Shenkang Hospital Development Center Three-year Funding for Major Clinical Research Projects (SHDC2020CR4062).

## Conflict of interest

The authors declare that the research was conducted in the absence of any commercial or financial relationships that could be construed as a potential conflict of interest.

## Publisher’s note

All claims expressed in this article are solely those of the authors and do not necessarily represent those of their affiliated organizations, or those of the publisher, the editors and the reviewers. Any product that may be evaluated in this article, or claim that may be made by its manufacturer, is not guaranteed or endorsed by the publisher.

## Supplementary material

The Supplementary material for this article can be found online at: https://www.frontiersin.org/articles/10.3389/fnagi.2023.1108561/full#supplementary-material

## Footnotes

^1^ http://bibliometric.com/

## Abbreviations

3D: Three-dimensional
AD: Alzheimer’s disease
BBB: Blood–brain barrier
EC: Endothelial cell
iPSCs: Induced pluripotent stem cells
NF-κB: Nuclear factor-κB
NO: Nitric oxide
PD: Parkinson’s disease
