# Supplementary material for: Bibliometric analysis of research topics on blood–brain barrier breakdown and cognitive function over the last two decades (2000–2021)
Source: Front Aging Neurosci. 2023 May 30;15:1108561. doi: 10.3389/fnagi.2023.1108561 (PMC10268002; doi:10.3389/fnagi.2023.1108561)
Supplement: Supplementary file 1 [file Table_1.pdf]

Table 1. Top 5 institutions with the highest number of publications

Rank	Institution	Country	Total publications
1	John Hopkins University	the United States	67
2	University of Kentucky	the United States	62
3	University of California San Francisco	the United States	60
4	Chinese Academy of Sciences	China	59
5	Fudan University	China	56
