# Supplementary material for: Bibliometric analysis of research topics on blood–brain barrier breakdown and cognitive function over the last two decades (2000–2021)
Source: Front Aging Neurosci. 2023 May 30;15:1108561. doi: 10.3389/fnagi.2023.1108561 (PMC10268002; doi:10.3389/fnagi.2023.1108561)
Supplement: Supplementary file 2 [file Table_2.pdf]

Table 2. Top 10 active journals with the highest number of total citations that published articles in BBB-cognition research (sorted by count).

| Rank | Journal title | Total citations | Average citation per paper | Impact factor （2021） | Country | JCR |
|---|---|---|---|---|---|---|
| 1 | Journal of Alzheimers Disease | 561 | 2.74 | 4.47 | Netherlands | Q2 |
| 2 | Journal of Neuroscience | 444 | 6.25 | 6.17 | the United States | Q1 |
| 3 | Nature Medicine | 422 | 46.89 | 53.44 | the United States | Q1 |
| 4 | Neurobiology of Aging | 422 | 46.89 | 4.67 | England | Q2 |
| 5 | Journal of Clinical Investigation | 318 | 35.33 | 14.81 | the United States | Q1 |
| 6 | Neuron | 311 | 51.83 | 17.17 | the United States | Q1 |
| 7 | PLOS ONE | 291 | 2.17 | 3.24 | the United States | Q2 |
| 8 | Proceedings of the National Academy of Sciences of the United States Of America | 290 | 6.59 | 11.21 | the United States | Q1 |
| 9 | Journal of Cerebral Blood Flow and Metabolism | 279 | 4.73 | 6.20 | the United States | Q1 |

| | | | | | | |
|---|---|---|---|---|---|---|
| 10 | Brain Research | 245 | 3.18 | 3.25 | Netherlands | Q2 |
