# Supplementary material for: Bibliometric analysis of research topics on blood–brain barrier breakdown and cognitive function over the last two decades (2000–2021)
Source: Front Aging Neurosci. 2023 May 30;15:1108561. doi: 10.3389/fnagi.2023.1108561 (PMC10268002; doi:10.3389/fnagi.2023.1108561)
Supplement: Supplementary file 3 [file Table_3.pdf]

Table 3. Top 5 most cited authors in BBB-cognition field.

Rank	Author	Institution	Country
1	Berislav V. Zlokovic	Department of Neurological Surgery at the Children’s Hospital in Los Angeles	the United States
2	Dennis J Selkoe	Department of Neurology, Brigham and Women's Hospital and Harvard Medical School	the United States
3	William A Banks	Division of Gerontology and Geriatric Medicine, Department of Medicine, University of Washington School of Medicine	the United States
4	N Joan Abbott	King's College London, Blood-Brain Barrier Group, Pharmaceutical Science Division, Hodgkin Building, Guy's Campus	the United Kingdom
5	Rashid Deane	Center for Translational Neuromedicine, Department of Neurosurgery, University of Rochester Medical Center	the United States
